# Unqualified Practitioners and “Criminal Negligence”

**Published:** 1902-12

**Authors:** 


### Unqualified Practitioners and "Criminal Negligence."

The unqualified man who practised at Willesden, assuming the names of qualified practitioners resident in the Isle of Wight and elsewhere, was on October 29, 1902, convicted of manslaughter at the Central Criminal Court, and sentenced to five years' penal servitude. The charge of perjury, founded on the fact that he had sworn at the inquest that he was a registered medical practitioner, was not proceeded with. The case illustrates the extraordinary ease with which, under the existing state of the law, a person entirely unacquainted with medicine may practice with impunity until he is tripped up by the consequences of his gross ignorance in dealing with a serious case. It will be particularly remembered, owing to the observations made by the judge in his summing-up and his direction to the jury. It has been previously held by the judges that an unqualified and a qualified practitioner were on exactly the same plane in respect of charges of criminal negligence, and that the question of legal qualification and registration was practically immaterial to the issue. Mr. Justice Bigham, as will be seen from the full report of his summing up, which is published at page 1475, has gone a good deal beyond this. He gave it as his opinion that a man like the prisoner to hold himself out as having medical skill was in itself an act of gross negligence, and instructed the jury to take that as a direction from him upon the point. By this ruling the assumption of a medical qualification, coupled with the assumption of medical skill represented by such medical qualifications, is proof of culpable neglect. The importance of this will at once be perceived, inasmuch as it will render the position of the unqualified practitioner in the future much more difficult, and will serve to protect the statutory privileges conferred upon the medical profession. That it is in accord with public policy must be equally obvious, since it will increase the degree of public protection against impostors which it is the object of the medical acts to create.—*British Medical Journal*, November, 1902.

